# An Analysis and Literature Review of a Family Case of Acute Intermittent Porphyria With Initial Symptoms of Epileptic Seizure

**DOI:** 10.7759/cureus.45736

**Published:** 2023-09-21

**Authors:** Jinxing Lai, Zhenzhou Zhong, Zhaohui Lai, Xianghong Liu

**Affiliations:** 1 Department of Neurology, The Affiliated Ganzhou Hospital of Nanchang University, Ganzhou, CHN; 2 Emergency Department, The Affiliated Ganzhou Hospital of Nanchang University, Ganzhou, CHN

**Keywords:** gene mutation, family case report, seizure, acute intermittent porphyria, porphyria

## Abstract

Acute intermittent porphyria (AIP) is the most common form of acute porphyria and is characterized by acute onset and recurrent episodes. Clinical presentation frequently initiates with gastrointestinal symptoms and is often misdiagnosed or delayed secondary to nonspecific symptoms. Acute porphyria with epilepsy as the primary symptom is a very unusual or unexpected manifestation. This family case found an unexpected association between acute porphyria and seizures. This patient is a 33-year-old woman whose initial symptom was symptomatic epilepsy, followed by significant abdominal pain. After excluding infection, immunity, and other factors, whole exome sequencing analysis showed the presence of c.22dupG mutation in the HMBS gene and the patient was finally diagnosed with AIP. Her symptoms significantly improved after receiving high-glucose and high-carbohydrate load treatment. This case report is rare and suggests that for patients who experience epileptic seizures coupled with complaints related to the abdomen, the possibility of porphyria should be specially considered in the differential diagnosis.

## Introduction

Porphyria is a metabolic disorder in humans that is characterized by various modes of inheritance and clinical manifestations and is caused by an enzymatic deficiency in the heme biosynthetic pathway [1]. Acute intermittent porphyria (AIP) is a subgroup of porphyria with attacks typified by abdominal pain, neurological symptoms, and psychiatric disorders. In severe cases, it can lead to respiratory paralysis and coma [2,3]. Abdominal pain in AIP is caused by spasms of smooth muscle caused by preganglionic fibre damage and can also cause constipation and bloating. Approximately 10-40% of patients may manifest signs of peripheral neuropathy during episodes of AIP. Notably, the severity of affliction is typically less pronounced in these patients [4]. AIP is the most common form of hereditary hepatic porphyria in adolescent females and is triggered by factors such as alcohol consumption, low-carbohydrate diets, menstruation, infections, surgical procedures, and various porphyrinogenic drugs. Patients with latent AIP may have no or only mild clinical symptoms, and biochemical test results may be normal [5]. Despite its rarity, the disease can be diagnosed by noting a decrease in the levels of porphobilinogen in red blood cells. However, while autonomic changes and seizures are highly indicative, misdiagnosis is common and confirmation requires genetic testing [6,7].

We report a case of a 33-year-old female diagnosed with AIP in whom genetic testing revealed a new heterozygous mutation in the hydroxymethylbilane synthase (HMBS) gene (NM_000190.4:c.22dupG). This mutation was also found in her relatives. These findings can assist in protecting probands and carriers of the variant from life-threatening acute attacks. This case contributes to the increasing understanding of AIP diagnoses.

## Case presentation

**Figure 1:**
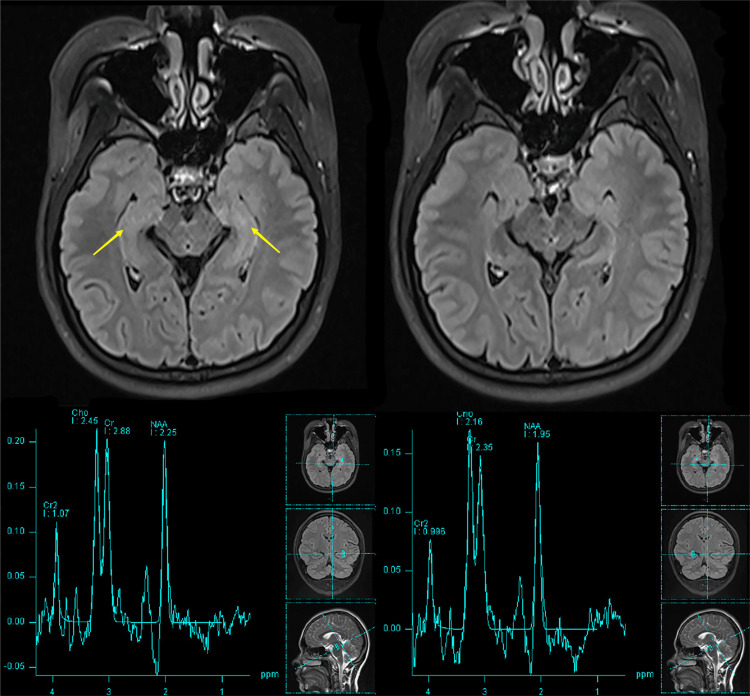
MR spectroscopy indicated bilateral hippocampal neuronal dysfunction

**Figure 2:**
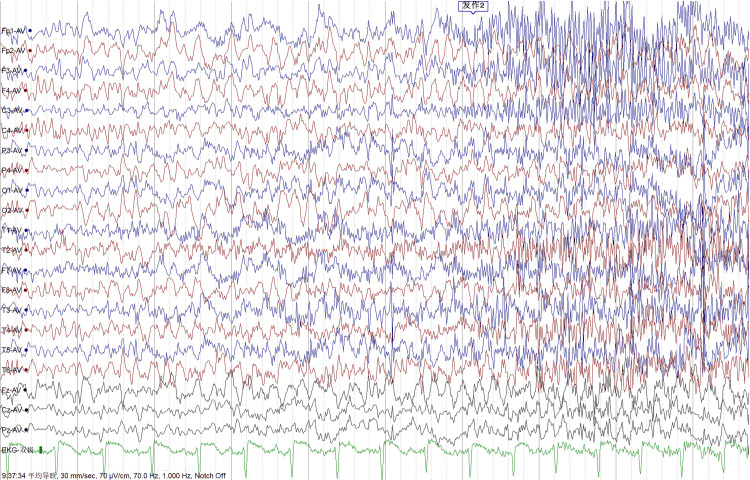
Electroencephalogram showing partial seizures followed by generalized seizures

The patient was a 33-year-old female who first sought treatment at the Affiliated Ganzhou Hospital of Nanchang University due to "episodic limb convulsions for one day". She reported a spontaneous onset of twitching at the left corner of her mouth during rest, presenting with pallor and rigid twitching of her left upper limb. The symptoms lasted for approximately one minute before easing, and they recurred several times over the span of two hours without any loss of consciousness. Upon further inquiry into her medical history, she revealed a similar episode 10 days prior. Considering the possibility of epilepsy, she was admitted. To rule out autoimmune etiologies, tests were conducted for central nervous system (CNS) demyelinating antibodies and autoimmune encephalitis antibodies in the cerebrospinal fluid, but no positive results were found. Her cranial magnetic resonance imaging (MRI) scans were normal, but MR spectroscopy indicated bilateral hippocampal neuronal dysfunction (Figure 1). Long-term video electroencephalogram showed no significant abnormal brain waves during the interictal period, while partial seizures followed by generalized seizures were noted during the ictal period, consistent with the electroencephalogram (EEG) progression of epileptic seizures (Figure 2). Given the clinical manifestations, a tentative diagnosis of viral encephalitis and status epilepticus was made, and she was treated with acyclovir（0.5g q8h VD, midazolam(5mg ST IV), levetiracetam(0.5g BID PO). Her condition stabilized after the treatment. She was discharged 14 days later and was advised to continue taking her medications orally.

She presented again for medical evaluation 10 days post-discharge, complaining of bilateral lumbar pain persisting for over 10 days. She reported the onset of this pain in the bilateral lumbar region, without any obvious cause, more than 10 days ago. The pain was described as intense and unbearable, abdominal examination revealed mild tenderness in the mid-abdomen with no rebound tenderness. An emergency abdominal computerized tomography (CT) scan was performed, which did not reveal any significant findings. Upon examination, she was found to be positive for antinuclear antibodies (ANAs) at a titer of 1:100. Complement C3 was measured at 59 mg/dL, and complement C4 was measured at 16.1 mg/dL. Twenty-four-hour urinary protein quantification was negative. She was also positive for perinuclear antineutrophil cytoplasmic antibodies (P-ANCA) at a titer of 1:100. Additionally, anti-histone antibody (AHA), anti-cyclic citrullinated peptide (CCP) antibody, and anti-β2 glycoprotein 1 (β2-GP1) antibody were also detected as positive. Lupus anticoagulant (LA) was negative. A lumbar puncture was conducted, which showed predominantly normal cerebrospinal fluid (CSF) biochemical profiles. Both the results of cerebrospinal fluid-next-generation sequencing (CSF-NGS) and the autoantibody assays were negative. In combination with clinical symptoms, she was provisionally diagnosed with CNS vasculitis, autoimmune encephalitis (unknown antibodies) and epilepsy. She was continued on sedative and antiepileptic medications (midazolam 5mg ST IV, levetiracetam 0.75g BID PO), along with a low dose of steroids(methylprednisolone sodium succinate 80mg VD QD). Her symptoms improved, and she was subsequently discharged from the hospital.

**Figure 3:**
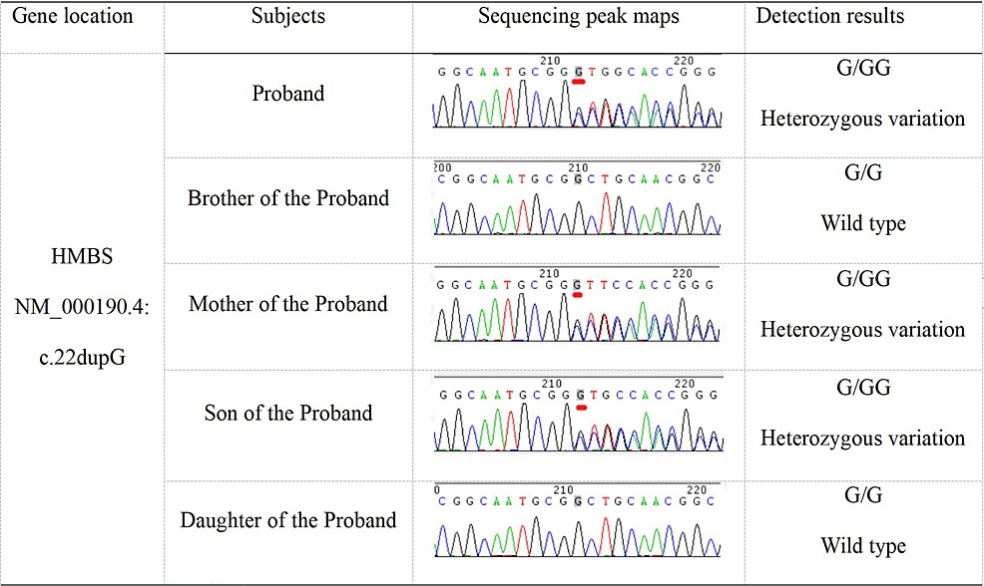
Variant site validation results

During her second hospitalization, a whole exome sequencing analysis was conducted, indicating a c.22dupG mutation in the HMBS gene. Concurrently, an antinuclear antibody profile test was performed, showing ANA positivity (1:80), IF-ANCA positivity, rheumatoid factor (RF) 125.0 IU/mL, anti-CCP positivity, and LA and anti-phospholipid antibody negativity. She was readmitted for treatment with a tentative diagnosis of AIP, not excluding the possibility of connective tissue disease. Upon admission, the uroporphyrin test results were negative, the uroporphyrinogen test results were positive, and several RF antibodies were positive. Family verification revealed that the proband's mother and the proband's son carried the HMBS gene NM_000190.4:c.22dupG mutation. Due to differences in the mutation rate of the genetic variant, her mother and son have not developed the disease temporarily. The proband's brother and daughter did not carry this mutation. The results of the mutation verification are shown in Figure 3. After admission, she was treated with oral high-glucose, high-carbohydrate load supplemented with sodium and potassium, and electrolytes were monitored. In addition, she was continued on antiepileptic drugs and liver protection treatment. She exhibited a substantial improvement in symptoms after the above treatment. After her condition stabilized, she was discharged.

## Discussion

AIP is the most common acute porphyria worldwide, with an estimated prevalence of 5-10 cases per 100,000 [8]. AIP is an autosomal dominant disorder that is more frequently seen in females (due to hormonal influences) and often occurs after puberty [9]. Symptoms often present in discrete episodes, improving or recurring within a few days, but approximately 90% of genetic carriers may remain asymptomatic throughout their lives [7]. AIP presents with acute porphyria symptoms, most commonly affecting the gastrointestinal and nervous systems, including abdominal and limb pain, with no skin manifestations. The initial symptom of AIP often manifests as unexplainable severe abdominal pain accompanied by nausea. Acute attacks of AIP are usually triggered by certain factors [10], including medications, infections, fasting, alcohol abuse, and glucocorticoid steroids. During acute episodes, patients' urine may turn dark brownish-red due to the presence of porphyrins (which are pale red) and porphobilinogen (a brown polymer degradation product of colourless porphyrin precursors) [11]. The symptoms and signs of AIP may involve multiple organ systems of the body, with the timing and severity of attacks varying among patients. In certain cases, particularly in the absence of timely diagnosis and proper treatment, AIP may lead to life-threatening complications [12]. It is noteworthy that there is a high degree of variability in AIP symptoms, and all the mentioned symptoms may not be present. Patients with AIP typically recover within a few days, but if an acute attack is not promptly diagnosed and treated, the recovery time may be prolonged, extending to several weeks or even months. Most patients do not manifest any symptoms in the interictal period [12].

Given the rarity of AIP and the resemblance of its symptoms and signs to other common diseases, it is often misdiagnosed. Elevated levels of δ-aminolevulinic acid (δ-ALA) and porphobilinogen (PBG) in urine are among the common indicators of acute porphyria [13]. If urinary PBG excretion is increased, further testing (faecal and blood porphyrin determination) is necessary to distinguish between AIP, variegate porphyria, or hereditary coproporphyria [14]. Now, ample evidence suggests that once urinary PBG excretion increases in AIP, it takes many years to return to normal. Hence, increased urinary PBG excretion in a patient with a history of AIP does not indicate an acute attack [15]. Whether in acute or chronic forms of AIP, the cornerstone of treatment includes inhibiting the production of ALA and PBG. The main approach during acute episodes of AIP continues to be the cessation of porphyrinogenic drugs, ample caloric support, heme therapy, and symptomatic treatment [16]. Acute AIP attacks usually require hospitalization, requiring drugs to treat pain, nausea, and vomiting, intravenous fluids, and close observation. Suppression of disease activity can be aided by intravenous or oral glucose and other carbohydrates, which are part of the initial treatment [17]. High blood sugar can interfere with ALAS1 transcription, and if the patient has mild symptoms (e.g., no weakness, no vomiting, or no hyponatremia), a high-carbohydrate diet may be considered 48 hours before starting specific therapy [18]. If the disease is severe, intravenous glucose (300 ~ 500 g/d) is often given. If the condition persists in affecting eating, a more comprehensive parenteral nutrition regimen is needed. The current consensus is that glucose infusion regimens are readily available but relatively weak, and because dilution of large amounts of free water can exacerbate hyponatremia, careful monitoring of electrolytes should be taken when used [19]. If the patient's symptoms have not improved within 36 hours, heme can be administered intravenously [20]. Heme not only downregulates ALAS1 transcription through negative feedback but also reduces ALAS1 levels in the liver by interfering with mRNA stability or blocking mature enzymes from entering mitochondria. However, there is obvious controversy over whether to carry out heme preventive therapy, not only because intravenous heme has side effects such as venous thrombosis, coagulation dysfunction, liver fibrillation, and iron overload but also because repeated intravenous heme treatment will reduce the efficacy, possibly because heme causes chronic inflammatory liver disease, thereby gradually activating heme oxygenase 1 expression but inducing high expression of ALAS1 [21]. In the treatment of an attack, attention should be given to electrolyte balance. The use of harmful drugs should be discontinued. Many antiepileptic drugs can induce acute seizures in AIP, and if the patient develops seizures, they can be controlled with diazepam, clonazepam, or magnesium sulfate, and barbiturates are contraindicated [22]. The treatment of AIP also includes medications to treat specific symptoms, such as certain analgesics, anti-anxiety medications, antihypertensive drugs, and medications to treat nausea and vomiting, tachycardia, or restlessness [23]. For patients with recurrent attacks, approximately 5% of patients have recurrent acute porphyria. Patients may experience recurrent acute visceral episodes, chronic symptoms, and long-term complications [24]. Low-dose gonadotropin-releasing hormone analogues can be used to treat menstruation-related acute porphyria, but the exact efficacy is highly controversial. Prophylactic heme transfusions are beneficial, but complications occur, and repeated infusions are less effective. Liver transplantation and hepatocyte transplantation are also used to treat patients who have failed other treatments, but there are many comorbidities, and lifelong immunosuppressive therapy is required [25]. 

Looking back at the diagnosis and treatment process for this patient, epilepsy was the initial symptom, and all examinations did not indicate any AIP-related changes. After sedation and anti-epileptic treatment, the symptoms were alleviated. However, 11 days after discharge, the patient was readmitted with bilateral lower back pain for 10 days. The medical history indicated severe pain accompanied by nausea and decreased appetite, with no notable abdominal pain. The presentation of epilepsy combined with unexplained lower back pain in this patient provides suggestive indications for the diagnosis of AIP. Upon admission, further comprehensive examinations, such as abdominal, cranial, and autoimmune antibody-related tests, were conducted, which led to additional diagnoses of CNS vasculitis and autoimmune encephalitis. Symptomatic treatment was continued. During treatment, the patient also presented with lower limb weakness, but the symptoms were not prominent and did not receive due attention. To further clarify the cause of the disease, whole-exome sequencing was performed. The results suggested a c.22dupG mutation in the HMBS gene, raising the consideration for a diagnosis of AIP. Subsequent urinary tests revealed negative uroporphyrin but positive PBG, confirming the AIP diagnosis. High-sugar and high-carbohydrate load treatment was administered, alleviating back pain and restoring normal muscle strength in the lower limbs. Notably, in the process of diagnosis and treatment, a detailed history failed to identify the specific triggers for this patient's episodes.

There have been research reports suggesting that the triad of hyponatremia, intermittent epileptic seizures, and abdominal pain should raise suspicion of porphyria [10]. Abdominal pain is the most common clinical manifestation and the first symptom in the onset of AIP and is considered the signal of acute onset of AIP. A 2020 large-scale population-based study in the United States showed that approximately 1/4 of patients with a history of abdominal pain had symptoms similar to AIP [26]. The clinical symptoms of AIP patients reported in China are mainly abdominal pain, most of which is severe abdominal pain, often accompanied by digestive tract symptoms such as nausea, vomiting, and constipation [27]. Therefore, patients with AIP are often misdiagnosed with intestinal obstruction, acute gastroenteritis, appendicitis, etc., and treatment is delayed. Moreover, these gastrointestinal symptoms often cause decreased appetite and impaired absorption of energy intake, resulting in negative energy balance, which in turn further exacerbates the onset of AIP. Neuropsychiatric symptoms are another major feature of acute episodes of AIP, which can be divided into central nervous symptoms (seizures, impaired consciousness, coma), peripheral neurological symptoms (numbness, pain, fatigue in the extremities, etc.), autonomic symptoms (hypertension, tachycardia, hyperhidrosis, etc.) and psychiatric symptoms (depression, anxiety, hallucinations, etc.) [4]. At present, the most common neurological symptoms reported in AIP patients in China are impaired consciousness, seizures, hypertension and tachycardia. Some patients with AIP may present with psychiatric symptoms such as depression and anxiety, which in turn can be used as a trigger for an acute onset of AIP to exacerbate the condition [28].

In this case, the patient presented with symptomatic epilepsy as the initial symptom, followed by symptoms such as lumbar pain with nausea and weakness of the lower limbs. For early diagnosis, Kazamel et al. recommended conducting urinary porphobilinogen tests for any unexplained recurrent severe acute abdominal pain, especially in patients with concurrent neuropathic pain, hyponatremia, autonomic dysfunction, or encephalopathy [29]. The symptoms of the present case bear similarities to the case described by Subashri et al. [30]. The patient was admitted for treatment with a chief complaint of "generalized spasticity." However, upon further inquiry into the medical history, intermittent abdominal pain and significant electrolyte imbalances were found. There are differences in the timing and type of symptoms compared to the present case, but both cases did not present residual neurological dysfunction after treatment [30]. Kumar reported a case presenting solely with psychiatric symptoms, lacking any accompanying physical features, such as abdominal pain or neuropathy, which did not completely rule out the diagnosis [31]. The low penetrance of AIP significantly underestimates the prevalence of AIP. Every individual with an HMBS mutation is likely to have an acute onset of AIP, and AIP, as a recurrent disease, may lead to irreversible chronic complications such as hepatocellular carcinoma and renal insufficiency [32]. Currently, the widespread adoption of diagnostic methods, utilization of hematin preparations, and prevention of acute attacks have significantly reduced the mortality rate. However, if the diagnosis of AIP is delayed, the mortality rate of acute attacks is 5-20%, which is still at a high level [33].

It is worth noting that identifying predisposing factors plays a crucial role in preventing the acute onset of AIP. Actively control infection, maintain good living habits to ensure sufficient energy intake, quit smoking and alcohol, and try to avoid emotional agitation. For the treatment of obese patients, a certain amount of carbohydrates should be added to the diet to reduce weight at a relatively slow rate. Because seizures are related to the menstrual cycle, GnRH-a, such as triptorelin acetate, can prevent seizures. Elevated estrogen levels in pregnant women can induce acute attacks of AIP, and ALA can be toxic to the fetal brain through the placenta, so patients with AIP are recommended to become pregnant only after at least two years in remission [34]. At the same time, the family members of minors should actively make relevant genetic diagnoses because it can reduce the probability of acute attack in patients who have been diagnosed with AIP in the asymptomatic stage to 5% [35]. Therefore, early detection, early diagnosis, early treatment, and identification of the relevant aggravating factors should be performed to avoid the serious adverse consequences caused by acute attacks of AIP.

## Conclusions

AIP is a motor neuropathy characterized by diverse clinical manifestations, resembling various neurological disorders. In the differential diagnosis of patients presenting with nonspecific abdominal and neuropsychiatric symptoms, consideration should be given to AIP. Early diagnosis through appropriate management can significantly improve the prognosis of patients.

## References

[1] Luvai A, Mbagaya W, Narayanan D (2015). Hepatocellular carcinoma in variegate porphyria: a case report and literature review. Ann Clin Biochem.

[2] Spiritos Z, Salvador S, Mosquera D, Wilder J (2019). Acute intermittent porphyria: current perspectives and case presentation. Ther Clin Risk Manag.

[3] Phillips JD (2019). Heme biosynthesis and the porphyrias. Mol Genet Metab.

[4] Wylie K, Testai FD (2022). Neurological manifestations of acute porphyrias. Curr Neurol Neurosci Rep.

[5] Bonkovsky HL, Maddukuri VC, Yazici C (2014). Acute porphyrias in the USA: features of 108 subjects from porphyrias consortium. Am J Med.

[6] Hu Y, Li W, Kang N (2022). Identification and molecular analysis of 17 novel variants of hydroxymethylbilane synthase in Chinese patients with acute intermittent porphyria. Clin Genet.

[7] Pischik E, Kauppinen R (2015). An update of clinical management of acute intermittent porphyria. Appl Clin Genet.

[8] Bissell DM, Wang B (2015). Acute Hepatic Porphyria. J Clin Transl Hepatol.

[9] Yasuda M, Chen B, Desnick RJ (2019). Recent advances on porphyria genetics: Inheritance, penetrance &amp; molecular heterogeneity, including new modifying/causative genes. Mol Genet Metab.

[10] Stein PE, Badminton MN, Rees DC (2017). Update review of the acute porphyrias. Br J Haematol.

[11] Marsden JT, Rees DC (2014). Urinary excretion of porphyrins, porphobilinogen and δ-aminolaevulinic acid following an attack of acute intermittent porphyria. J Clin Pathol.

[12] Dhital R, Basnet S, Poudel DR, Bhusal KR (2017). Acute intermittent porphyria: a test of clinical acumen. J Community Hosp Intern Med Perspect.

[13] Ma Y, Teng Q, Zhang Y, Zhang S (2020). Acute intermittent porphyria: focus on possible mechanisms of acute and chronic manifestations. Intractable Rare Dis Res.

[14] Stölzel U, Doss MO, Schuppan D (2019). Clinical guide and update on porphyrias. Gastroenterology.

[15] Gomez-Gomez A, Marcos J, Aguilera P, To-Figueras J, Pozo OJ (2017). Comprehensive analysis of the tryptophan metabolome in urine of patients with acute intermittent porphyria. J Chromatogr B Analyt Technol Biomed Life Sci.

[16] Kizilaslan EZ, Ghadge NM, Martinez A (2023). Acute intermittent porphyria’s symptoms and management: a narrative review. Cureus.

[17] Di Pierro E, Granata F (2020). Nutrients and porphyria: An intriguing crosstalk. Int J Mol Sci.

[18] Solares I, Jericó D, Córdoba KM, Morales-Conejo M, Ena J, Enríquez de Salamanca R, Fontanellas A (2022). Understanding carbohydrate metabolism and insulin resistance in acute intermittent porphyria. Int J Mol Sci.

[19] Anderson KE (2019). Acute hepatic porphyrias: Current diagnosis & management. Mol Genet Metab.

[20] Longo M, Paolini E, Meroni M, Dongiovanni P (2022). Cutting-edge therapies and novel strategies for acute intermittent porphyria: step-by-step towards the solution. Biomedicines.

[21] Granata F, Brancaleoni V, Barman-Aksözen J (2022). Heme biosynthetic gene expression analysis with dPCR in erythropoietic protoporphyria patients. Front Physiol.

[22] Zheng X, Liu X, Wang Y (2018). Acute intermittent porphyria presenting with seizures and posterior reversible encephalopathy syndrome: Two case reports and a literature review. Medicine (Baltimore).

[23] Neeleman RA, Wagenmakers MA, Koole-Lesuis RH, Mijnhout GS, Wilson JH, Friesema EC, Langendonk JG (2018). Medical and financial burden of acute intermittent porphyria. J Inherit Metab Dis.

[24] Gouya L, Ventura P, Balwani M (2020). EXPLORE: A prospective, multinational, natural history study of patients with acute hepatic porphyria with recurrent attacks. Hepatology.

[25] Schulenburg-Brand D, Gardiner T, Guppy S (2017). An audit of the use of Gonadorelin analogues to prevent recurrent acute symptoms in patients with acute porphyria in the United Kingdom. JIMD Rep.

[26] Ramanujam VS, Anderson KE (2015). Porphyria diagnostics-Part 1: a brief overview of the porphyrias. Curr Protoc Hum Genet.

[27] Yang J, Zhu T, Zhao Y, Yu X, Zhu H, Jiang Y, Li X (2018). Acute intermittent porphyria in the north of China: the acute attack effect on quality of life and psychological condition. Biomed Res Int.

[28] Wang Y, Chen XY, Li Y, Dong XH, Xu F (2019). Clinical characteristics of 50 patients with acute intermittent porphyria [article in Chinese]. Zhonghua Nei Ke Za Zhi.

[29] Kazamel M, Pischik E, Desnick RJ (2022). Pain in acute hepatic porphyrias: Updates on pathophysiology and management. Front Neurol.

[30] Subashri M, Prasad ND, Fernando E, Raj YT (2021). Recurrent seizures in an adolescent female-a daunting puzzle. Indian J Nephrol.

[31] Kumar B (2012). Acute intermittent porphyria presenting solely with psychosis: a case report and discussion. Psychosomatics.

[32] Ghosh R, León-Ruiz M, Singh Sardar S (2022). A novel heterozygous mutation in the hydroxymethylbilane synthase gene in a case with acute intermittent porphyria. Qatar Med J.

[33] Zhao L, Wang X, Zhang X, Liu X, Ma N, Zhang Y, Zhang S (2020). Therapeutic strategies for acute intermittent porphyria. Intractable Rare Dis Res.

[34] Innala E, Bäckström T, Bixo M, Andersson C (2010). Evaluation of gonadotropin-releasing hormone agonist treatment for prevention of menstrual-related attacks in acute porphyria. Acta Obstet Gynecol Scand.

[35] Kuo HC, Ro LS, Jung SM, Huang CC, Chu CC (2016). Porphyric neuropathies in an acute intermittent porphyria family. Neuropathology.

